# The Impact of Fibromyalgia in Spondyloarthritis: From Classification Criteria to Outcome Measures

**DOI:** 10.3389/fmed.2018.00290

**Published:** 2018-10-24

**Authors:** Alessia Alunno, Francesco Carubbi, Simon Stones, Roberto Gerli, Roberto Giacomelli, Xenofon Baraliakos

**Affiliations:** ^1^Rheumatology Unit, Department of Medicine, University of Perugia, Perugia, Italy; ^2^Rheumatology Unit, Department of Biotechnological and Applied Clinical Sciences, University of L'Aquila, L'Aquila, Italy; ^3^ASL1 Avezzano-Sulmona-L'Aquila, L'Aquila, Italy; ^4^School of Healthcare, University of Leeds, Leeds, United Kingdom; ^5^Fibromyalgia Action UK, Paisley, United Kingdom; ^6^Rheumazentrum Ruhrgebiet Herne, Ruhr University of Bochum, Bochum, Germany

**Keywords:** spondyarthropathies (SpA), fibromyaglia, back pain, enthesitis, imaging

## Abstract

The term spondyloarthritis (SpA) encompasses a broad clinical spectrum characterized by chronic inflammatory conditions affecting the sacroiliac joints, the spine but also peripheral joints and tendons and being additionally associated with the involvement of organs, such as bowel, eye and skin (1). Musculoskeletal pain is a key symptom in SpA. However, although low back pain and/or joint pain are characteristic for SpA, undifferentiated pain at different enthesial sites may also be a concomitant or even the first clinical presentation in some patients (2). In addition, fatigue is another important symptom often reported by patients with SpA, which substantially affects the quality of life (QoL) (3). Fibromyalgia (FM) is the most common diagnosis in patients complaining of chronic diffuse pain with fatigue and may occur alone or in association with chronic inflammatory diseases (4). The prevalence of FM ranges from 2 to 8% in the general population and it can reach up to over 50% in patients with other rheumatic and musculoskeletal diseases (RMDs) (5–7). FM has been identified as the most disabling RMD, based on the patients' perception that their medical condition is not properly recognized (8). This is also due to the poor knowledge about its pathogenesis, and therefore the lack of reliable biomarkers reveals a major unmet need requiring to be addressed in further research studies. Over the last decade, an increasing body of evidence described the impact of FM in SpA highlighting the pitfalls for correct classification, appropriate differential diagnosis and assessment of outcome measures in both conditions. The purpose of this review is to provide an overview of currently available data with regard to the coexistence and reciprocal features of FM and SpA.

## Criteria for FM and SpA: state of the art

### From psychogenic rheumatism to fibromyalgia syndrome

The term FM was initially proposed in 1975 (9) to replace the old term fibrositis and was officially approved by the American College of Rheumatology (ACR) Committee while developing the 1990 classification criteria (10). The evidence that widespread pain was a key symptom complained of patients and that trigger points were the most powerful discriminator between patients and controls, led to the inclusion of these two observations as classification criteria for FM (10). Compared to the first set of criteria, that were highly specific but poorly sensitive and therefore missed a consistent proportion of FM patients, the ACR 1990 criteria ensured a higher sensitivity. However, after 20 years and many debates on the same matter, it became evident that the 1990 criteria had similar major pitfalls. The lack of proper implementation of trigger point assessment in daily practice, mainly in primary care where most of FM diagnosis occur, led to symptom-based rather than criteria-based identification of patients. Furthermore, the lack of other key manifestations within the FM-related clinical spectrum, such as fatigue and cognitive symptoms, was a major argument underpinning the need of a new set of criteria. The Manchester criteria were published in 1996 and used a widespread pain diagram to establish the diagnosis, but still did not include other relevant domains in the clinical spectrum of FM (11). In 2003, the combination of a Regional Pain Scale (RPS) score, combined with a fatigue score on a visual analog scale (VAS), was proposed (the so-called “Survey criteria”) (12, 13) and a few years later, an ACR committee was convened to build the ACR 2010 preliminary diagnostic criteria. In these criteria, the widespread pain index (WPI) and the symptom severity (SS) scale score were included to encompass the wide clinical picture of FM. Using the 2010 criteria, patients may be diagnosed with FM if they fulfill specific cut-offs, both for the WPI and the SS concomitantly to symptoms lasting for ≥3 months and no other condition that could explain their pain (14). In 2011, a modified version of the 2010 criteria was proposed that relied on self-reported pain and a simplified self-reported version of somatic symptoms (15). Finally, criteria combining the 2010 and the modified 2011 set were released in 2016 (16). In parallel to the release of the 2010 diagnostic criteria, the French Rheumatic Pain Study Group (Cercled'Etude de la Douleur en Rhumatologie, CEDR) developed and validated a patient self-completed questionnaire for the detection of FM in patients with chronic widespread pain: the Fibromyalgia Rapid Screening Tool (FiRST) (17). A previous screening tool, to be employed in epidemiological studies, the London Fibromyalgia Epidemiology Study Screening Questionnaire (LFESSQ) was developed in 1999 (18) while the FiRST, owing to its briefness and good performance, was tailored to be a companion for clinicians in daily practice. FM should no longer be viewed merely as the physical manifestation of psychological distress but instead as a central and peripheral pain hypersensitivity syndrome that could be secondary, in some cases, to the chronic inflammation. On this basis, FM should be diagnosed based on positive criteria instead of by elimination and should be managed in its own right in patients with other comorbidities, including rheumatic diseases.

### The kaleidoscope of criteria for spondyloarthritis

Several sets of classification criteria have been proposed over the last decades not only to ensure the correct identification of patients displaying different clinical features, but also to acknowledge and incorporate the evolution of imaging techniques and the compelling need to reduce the diagnostic delay (19). The human leukocyte antigens (HLA) B27 gene, the natural function of which is to present intracellular peptides to cytotoxic T lymphocytes, is strongly associated with SpA (20). The prevalence of HLA-B27 in the general population ranges from around 2 to 6% (21, 22), while it reaches 94% in AS (20). However, in other phenotypes of the SpA spectrum, HLA-B27 can be detected in lower proportions ranging from 30 to 75% (20). Inflammatory back pain (IBP) is a hallmark of axial SpA (axSpA) and its features were defined in several sets of criteria (23–26). The insidious onset of back pain lasting for at least 3 months, associated with morning stiffness improving with exercise in an individual aged 40–45 years or below, represented the basis for subsequent criteria for axSpA including the Assessment of Spondyloarthritis International Society (ASAS) 2004 (24), the Berlin 2006 (25) and the ASAS 2009 (26). In parallel, Calin's definition was also incorporated in the framework of the 1966 New York criteria for ankylosing spondylitis (AS) (27) which modified version was released in 1984 (28). The first set of criteria encompassing the entire clinical spectrum of SpA was developed by Amor in 1990 (29), followed by the European Spondylarthropathy Study Group (ESSG) criteria of 1991 (30) and the ASAS (24, 26). With regard to PsA, the definition of Moll and Wright in 1973, evolved and culminated in the latest set of criteria which is currently used, was released in 2006 by the classification of psoriatic arthritis (CASPAR) study group (31). An open question fuelling the debate in the scientific community is the one relating to non-radiographic axial (nr-ax)-SpA (32). The ASAS 2009 criteria include a milestone in this regard, as the item “sacroiliitis on imaging” now includes not only definitive radiographic changes according to modified New York criteria but also acute bone marrow oedema on magnetic resonance imaging (MRI), highly suggestive of SpA. In this regard, the ASAS-OMERACT MRI group defined active sacroiliitis on MRI based on the kind (bone marrow oedema/osteitis, synovitis of sacroiliac joints, enthesitis or capsulitis) and extension of lesions (33).

## The performance of FM criteria in patients with concomitant SpA and vice-versa

The complexity of SpA and FM clinical pictures, along with the different classification criteria available, account for the difficulty to compare studies exploring FM prevalence in SpA and vice-versa. A recent systematic literature review and meta-analysis discussed 10 studies assessing FM prevalence in SpA using the ACR 1990 and/or 2010 classification as inclusion criteria (7) but, as summarized in Table 1, the actual number of available studies is higher. It is interesting to note that the large majority of studies, many of which were published after the release of the 2010 ACR criteria, still assessed FM with the 1990 ACR criteria (6, 34–40, 42, 44, 50), while only four used the 2010 ACR criteria (41, 47, 49, 52) and only two used both criteria (45, 48). Four studies explored the FiRST tool either alone (43) or in comparison to the ACR 1990 (46, 53) or ACR 2010 (51) criteria. With regard to AS, data resulting from over one thousand patients demonstrate a prevalence of FM ranging between 6.4 and 25% based on the ACR 1990 criteria (6, 34–36, 40, 46, 50). The only study using the ACR 2010 criteria, enrolling 211 AS patients, observed a prevalence of 12.7% (41), while the study using the FiRST tool reported a prevalence of 22.3% of FM in AS (compared to a prevalence of 6.4% with the ACR 1990 criteria) (46). In studies assessing FM in overall axSpA according to the ASAS criteria without prior FM diagnosis, the prevalence was 13.2% with the ACR 1990 criteria (48, 53), between 17.2 and 60% with the ACR 2010 criteria (41, 51) and 36% with the FiRST tool (51).

**Table 1:** Studies evaluating the prevalence of fibromyalgia in patients with spondyloarthritis.

**References**	**Disease**	**N°pts**	**SpA criteria**	**FM (1990 criteria)**	**FM (2010 criteria)**	**FM (FiRST)**
Aloush et al. (34)	AS	36	Mod. New York	25%	NA	NA
Almodóvar et al. (35)	AS	462	Mod. New York	4.1%	NA	NA
Azevedo et al. (36)	AS	71	Mod. New York	15%	NA	NA
Husted et al. (37)	PsA	390	CASPAR	27.2%	NA	NA
Roussou and Ciurtin (38)	SpA PsA	60 20	Calin and Berlin CASPAR	30% TeP criterion 50% TeP criterion	NA	NA
Husted et al. (39)	PsA	631	CASPAR	22%	NA	NA
Demirdal et al. (40)	AS	71	Mod. New York	16.9%	NA	NA
Salaffi et al. (41)	AS axSpA	211 191	Mod. New York ASAS	NA	12.7% 17.2%	NA
Haliloglu et al. (6)	AS	119	New York	12.6%	NA	NA
Graceffa et al. (42)	PsA	74	CASPAR	16%	NA	NA
Bello et al. (43)	SpA	185	ASAS	NA	NA	20.5%
Wach et al. (44)	axSpA pSpA	81 22	Amor and ASAS ESSG and ASAS	14.8% 27.3%	NA	NA
Brikman et al. (45)	PsA	73	CASPAR	8.2% TeP criterion 1.4% both criteria	16.4%	NA
Fan et al. (46)	AS nr-axSpA pSpA PsA	126 64 52	New York ASAS ASAS CASPAR	6.4% 23.9% NA 9.6%	NA	22.3% 36.4% NA 22.9%
Macfarlane et al. (47)	SpA	1,041 398 65	Mod. New York ASAS radiological ASAS clinical	NA	19.7% 25.2% 9.5	NA
Baraliakos et al. (48)	axSpA	200	ASAS	13.5%	24%	NA
Di Carlo et al. (49)	PsA	144	CASPAR	NA	18.8%	NA
Fan et al. (50)	AS PsA pSpA nr-axSpA	137 59 38 64	Mod. New York CASPAR ASAS ASAS	6.4% 9.6% 5.3% 23.9%	NA	NA
Dantu et al. (51)	axSpA	25	ASAS	NA	60%	36%
Dantu et al. (52)	axSpA	51	ASAS	NA	47%	NA
Moltó et al. (53)	axSpA	441 85	ASAS Any except ASAS	13.2 32.9	NA	38% 41.2%

*N°, number; Pts, patients; FM, fibromyalgia; AS, ankylosing spondilytis; SpA, spondyloarthritis; ax, axial; p, peripheral; PsA, psoriatic arthritis; nr, non-radiographic, TeP, tender points; NA, not assessed; ASAS, Assessment of Spondyloarthritis International Society; CASPAR, classification of psoriatic arthritis; ESSG, European Spondylarthropathy Study Group; FiRST, Fibromyalgia Rapid Screening Tool*.

The study considering nr-axSpA as a separate entity reported a prevalence of FM of 23.9% and 36.4% using the ACR 1990 criteria and the FiRST tool, respectively (46). In peripheral (pSpA) according to either ESSG or ASAS criteria the prevalence of FM was assessed only with the ACR 1990 criteria and resulted between 5.3 and 27.3% (44, 50). All available studies on PsA used the CASPAR criteria and reported a prevalence of FM ranging from 1.4 to 37.3% with the ACR 1990 criteria (37, 39, 42, 45, 46) and from 16.4 to 18.8% with the ACR 2010 criteria (45, 49). The main observation arising from the above mentioned studies is that, despite the pitfalls pointed out about the 1990 ACR criteria (the lack of their adequate implementation in clinical practice), and most importantly, the availability of a new and more recent set of criteria, these are still the most commonly employed for research purposes. It is therefore difficult to translate these results into clinical practice and, most importantly, to fully understand similarities and differences not only among the two sets of ACR criteria but also with recent tools, such as the FiRST.

When looking at how many patients with FM would fulfill SpA criteria, the number of related publications is much lower and, as a consequence, the results even less clear (Table 2). In one study conducted in patients diagnosed with FM according to the ACR 1990 criteria, only 10% fulfilled the ASAS criteria (56). Of these, 80% fulfilled the ASAS criteria based on MRI findings diagnostic of sacroiliitis and the remaining 20% fulfilled the criteria based on the presence of HLA–B27 and SpA features. The study by Rousseau et al explored how many patients with FM would fulfill three sets of clinical criteria for SpA according to the ACR 2010 criteria. Over 60% of FM patients fulfilled either the Calin or the Berlin criteria, while 23% fulfilled the clinical arm of the ASAS criteria (55). No data are available regarding the evidence of radiographic or MRI sacroiliitis. In another cohort of FM patients diagnosed according to the ACR 2010 criteria, only 2% of them fulfilled the ASAS criteria (48). Interestingly, such low prevalence could be explained by the prospective nature of the study in a real-life population of patients with axSpA. When assessing the subgroup of FM patients with HLA-B27 available, this proportion raised to 5% but still remained very much below the 23% observed by Roussou and Georgiou (55). Finally, when considering the Amor or ESSG criteria, they were fulfilled by almost half of FM patients (diagnosed with the ACR 1990 and 2010 criteria) (54). Of interest, the prevalence of radiographic sacroiliitis grade 2 or higher was similar in patients' subgroups fulfilling either ACR 1990, ACR 2010 or both set of criteria and was around 30%. Of those with sacroiliitis grade 1 or 2 at conventional radiograms, only 4% displayed sacroiliitis at MRI. These findings open an intriguing scenario for further discussion which also includes the relevance of imaging in shedding some light on the actual co-existence, with consequent therapeutic implications, of FM and SpA. Moreover, it should be kept into account that fatigue can be detected in up to half SpA patients (57). This can be due not only to disease activity but notably also to psychogenic factors, especially depression. Therefore, it is imperative to rule out whether we are dealing with SpA-related fatigue, psychogenic fatigue or an actual diagnosis of FM. Unfortunately, such distinction is not easy, therefore, the question remains on whether it came first the chicken or the egg.

**Table 2:** Studies evaluating the prevalence of spondyloarthritis in patients with fibromyalgia.

**References**	**N°pts**	**Criterion for FM diagnosis**	**SpA prevalence**	**nr-axSpA**	**Fulfilled criteria**
Kaşkari et al. (54)	41 15	ACR 1990 or 2010 ACR 1990 and 2010	31.7% 46.6%	NA	Amor and ESSG
Baraliakos et al. (48)	100	ACR 1990 and ACR 2010	2%	0	ASAS
Roussou and Georgiou (55)	95	ACR 2010	62% 69% 23%	NA	Calin Berlin ASAS
Ablin et al. (56)	99	ACR 1990	10%	2	ASAS

*N°, number; Pts, patients; FM, fibromyalgia; ACR, American College of Rheumatology; SpA, spondyloarthritis, nr-ax, non-radiographic axial; NA, not assessed; ASAS, Assessment of Spondyloarthritis International Society; ESSG, European Spondylarthropathy Study Group*.

## Imaging to differentiate SpA from FM

The observation that FM patients may fulfill SpA criteria and the need to differentiate active SpA features from FM for appropriate therapeutic management, point out the importance to complement clinical assessment with imaging techniques. With regard to IBP, radiographic sacroiliitis has long been a hallmark of the classification of AS, within the axSpA spectrum (58). However, this may not be present in early phases of the disease and some patients may never develop such major structural damage, as discussed above. In this scenario, and according to the ASAS 2009 criteria, the evaluation of sacroiliac joints through MRI is therefore crucial. A still open question is whether the ASAS classification criteria may lead to overinterpretation and misclassification, which in consequence, may induce overtreatment of patients with chronic back pain and FM, who may just show minor MRI changes or a positive HLA-B27 finding (48). An even more challenging scenario is SpA presenting as polyenthesitis without axial involvement and here the role of imaging is of utmost importance. Enthesitis, defined as inflammation of tendon, ligament, and joint capsule insertions to the bones, is a hallmark of SpA (59). Entheseal regions are well-innervated, but are completely avascular at attachment sites and have a low density of blood vessels in the adjacent ligaments and tendons (60). On this basis, although pain, tenderness and stiffness are its main clinical manifestations, in most cases enthesitis occurs without visible signs of clinical inflammation and without increased acute phase reactants (61). Moreover, many entheses are not clinically accessible, located deep inside the human body. Often, clinical examination of enthesitis is inconclusive and the decision to initiate or adapt therapy is difficult. Finally, these entheseal SpA may be extremely difficult to distinguish from FM. The enthesitis score used in SpA has been criticized owing to its lack of correlation with objective measures of enthesitis, such as MRI, and because it overlaps with the FM tender point score (38, 62). From these anatomical and clinical considerations, in order to differentiate SpA-related enthesitis and FM, we require sensitive and advanced imaging techniques. Radiography only detects changes at a relatively late stage of disease, such as periosteal bony apposition or bony erosions, and is inferior to MRI in the evaluation of the disease extent and severity (63, 64). Scintigraphy has been widely used in the past; however, due to its lack of specificity it has been replaced by MRI and ultrasound (US) (65, 66). Despite providing interesting preliminary results (67, 68), positron emission tomography (PET) does seem neither specific nor able to offer the same excellent spatial resolution as MRI, requiring further research in this field.

Musculoskeletal US with power Doppler (PDUS) is a valid and reliable means of evaluating SpA enthesitis (69). On this basis, PDUS may provide further helpful information for distinguishing enthesitis and FM. However, it is important to highlight that age-related changes may occur at normal entheses, particularly in patients with high body mass index (BMI), so it is possible that patients with FM may show some degree of entheseal change. Some studies suggest that abnormal vascularity at the entheseal level characterize SpA patients, even if asymptomatic, with respect to healthy controls (70–72), although the presence of Doppler positivity seems to have a low sensitivity (36%) (72). The range of enthesophytes, a chronic entheseal change, in healthy controls overlapped the ranges of patient with psoriasis and PsA (72). However, enthesophytes are not specific to enthesitis and may be related to mechanical forces. In a pilot study comparing PDUS-revealed entheseal involvement in patients with PsA and patients with FM, Marchesoni and colleagues concluded that PDUS assessment of peripheral entheses appears to be able to differentiate patients with PsA and patients with FM in terms of number and distribution of involved sites as well as presence of inflammatory changes (73). However, since the presence of at least 1 lesion at 1 site was quite common in patients with FM (80%), a cut-off point of ≥3 involved sites has been set, providing the greatest discriminating power in patients with PsA, who were the only patients with bony erosions. Inflammatory changes were present in the majority of PsA patients, and the involvement of plantar fascia and Achilles tendon was highly specific of PsA. Many entheseal sites are not accessible for US scans, and, especially in patients with chronic widespread pain, such as FM, their assessment requires an excessive amount of scanning time. MRI methods could overcome the difficulties in the assessment of deeply located enthesis. On MRI, enthesitis is associated with diffuse perientheseal soft tissue oedema that is more conspicuous than inflammation within the insertion, reflecting the greater vascularity of the peri-entheseal tissues (59). In this context, adjacent osteitis may be present, due to the consistent vascularity of the bone marrow. In their small prospective study, Godfrin and colleagues used technetium 99 m angioscintigraphy and MRI to assess entheses, focusing on sites where the pain was the most severe or where radionuclide scanning showed increased uptake, and a good agreement between radionuclide scanning and MRI was found (74). In particular, radionuclide scanning and MRI findings establish that isolated entheseal pain could indicate SpA, while both investigations are normal in patients with FM. In contrast to conventional MRI, which only covers one anatomical area in one scan, whole-body MRI (WB-MRI) allows assessment of all peripheral and axial joints and entheses from “head-to-toe” in one examination (75). The WB-MRI technique could ensure better assessment of disease extent and activity within one examination and therefore improve the detection of entheseal inflammation (76), unmasking clinically silent enthesitis (77), while being less obtrusive for patients. Enthesitis can be detected on WB-MRI with moderate agreement between WB-MRI and clinical examination, and, interestingly, patients had more enthesitis at clinical examination than on WB-MRI, whereas it was the opposite for healthy subjects. This may be explained by the presence of subclinical enthesitis, which may be related to other conditions inducing mechanical stress, such as high BMI or physical overuse (77). Although all the above progress is promising, WB-MRI technique is still under development and, before widespread clinical use, robust research and clarification concerning entheseal abnormalities found also in healthy subjects are needed (61). Although enthesitis is considered as the primary pathological process underling SpA, it is still difficult to differentiate whether patients have poly-enthesitis, FM or both, because the physical examination may be similar, and other clinical features, for example the response to NSAIDs, need to be taken into consideration. Furthermore, as mentioned above, the poorly understood pathophysiology of FM and the lack of reliable diagnostic biomarkers further complicate this matter. At present, imaging with PDUS scanning or selected MRI scanning of sites with predominant pain, seem to be the most useful tool to be used in this process. In the future, more advanced and sophisticated imaging techniques, such as PET in combination with CT or MRI, or whole-body MRI may be used for this purpose.

## FM impact on SpA outcome measures

Given the considerable coexistence of SpA and FM, another crucial point is the impact of the latter on SpA outcome measures and therefore the implications of therapeutic tailoring (78). Patients with AS and FM usually report significantly worse disease activity, function, global severity scores, and QoL (47). The AS-QoL score is higher in patients with SpA and FM (B, C, G) and several studies agree that patients with SpA and FM display also higher Bath ankylosing spondylitis disease activity index (BASDAI) scores (6, 34–36, 40, 47) and poorer function scores, as calculated with the Bath Ankylosing Spondylitis Functional Index (BASFI) (34–36, 43). In this regard, objective measures play a major role in discriminating the inflammatory SpA component of disease activity/damage assessment and minimize the interference of FM clinical features. This is supported by the evidence that the ankylosing spondylitis disease activity score (ASDAS)-C reactive protein (CRP), and to a lesser extent the ASDAS-erythrocyte sedimentation rate (ESR), where acute phase reactants are the item with the highest weight, are better discriminators compared to the BASDAI that only includes 10 mm visual analogic scales to be compiled by the patient (41, 44). Likewise, the Bath Ankylosing Spondylitis Radiology Index (BASRI), including AS-specific radiographic changes, is reduced in patients with comorbid FM and, in this regard, Almodovar and colleagues suggested a BASDAI/BASRI ratio equal or over 1.5 to identify patients at high probability to have FM associated with SpA (positive likelihood ratio of 3.1) (35). With regard to PsA, the composite psoriatic disease activity index (CPDAI) and the disease activity index for psoriatic arthritis (DAPSA) scores are higher in patients with concomitant FM (45). This also applies to a recently validated patient reported outcome, the Psoriatic Arthritis Impact of Disease (PsAID). In fact, FM is associated to the final PsAID score in PsA patients further underscoring its impact on SpA patient reported outcomes (PROs) (49).

When focusing on the involvement of enthesis, the enthesitis score used in SpA has been criticized owing to its lack of correlation with objective measures of enthesitis and overlaps with the FM tender point score (38, 62). In this regard, the Leeds enthesitis index (LEI) is significantly higher in PsA patients with concomitant FM.

Having concurrent FM is associated with a higher likelihood of having received biologic therapy in a study from the British Society for Rheumatology Biologics Register in Ankylosing Spondylitis (BSRBR-AS) (47). Bello et al. did not observe any differences with regard to the initiation of biologic treatment, but reported a shorter retention rate of the first biologic after 2 years in patients with SpA and FM compared to those with SpA only (43).

FM diagnosis could predict a lower probability to achieve minimal disease activity or remission in patients with PsA (42, 45) and for instance the LEI is the only variable not showing a significant reduction over time in patients treated with biologic agents (42, 45). Finally, comorbid FM has a negative impact on response to biologic therapies in axSpA (53). This does not reflect a real reduced efficacy due to concomitant FM but rather the objective and hard to overcome difficulty of patients to detach FM and SpA symptoms when reporting their assessment of disease activity through PROs. All these observations further support the importance of imaging techniques as companion in the diagnostic process to rule out FM in SpA patients and to outline of treatment strategies in order to avoid overestimation of SpA disease activity and therefore overtreatment.

## Concluding remarks

FM occurs in a significant proportion of SpA patients increasing the chronic symptom burden, reducing overall function, and worsening QoL. This has a dramatic impact not only on the individual but more in general on society in terms of decreased work productivity. While FM may coexist with SpA, disease activity measures that include subjective elements, such as pain and patient global reports may be overstated, even when more objective measures, such as swollen joint count or CRP suggest the achievement of remission or low disease activity. Therefore, PROs may be less valid and reliable as a true measure of inflammatory disease activity and to be used as a basis for therapeutic decision-making in clinical practice in some patients with long-standing disease, since they may lead to intensification of, or switching immunotherapy when it is not necessarily warranted.

On this basis, the coexistence of FM should be assessed in patients with SpA based on the clinician's judgment and the use of validated positive criteria/questionnaires, instead of by elimination criteria, with input from both patient and clinician. In addition, sensitive and advanced imaging techniques should be necessarily implemented to differentiate SpA inflammatory pain from FM pain, therefore establishing the most appropriate treatment approach in partnership with the patient.

## Author contributions

AA and FC conceived the idea of this review article. AA, FC, and SS prepared a first draft of the manuscript that was critically revised by XB, RGe, RGi. All authors approved the final version of the manuscript.

### Conflict of interest statement

The authors declare that the research was conducted in the absence of any commercial or financial relationships that could be construed as a potential conflict of interest.

## References

[1] Taurog JD Chhabra A Colbert RA. Ankylosing spondylitis and axial spondyloarthritis. N Engl J Med. (2016) 374:2563–74. 10.1056/NEJMra1406182 27355535

[2] Rojas-Vargas M Muñoz-Gomariz E Escudero A Font P Zarco P Almodovar R. Registro Español de Espondiloartritis de la Sociedad Española de Reumatología Working Group. First signs and symptoms of spondyloarthritis–data from an inception cohort with a disease course of two years or less (REGISPONSER-Early). Rheumatology (Oxford) (2009) 48:404–9 10.1093/rheumatology/ken506 19208685 PMC2656634

[3] Wendling D Prati C Spondyloarthritis and fibromyalgia: interfering association or differential diagnosis? Clin Rheumatol. (2016) 35:2141–3. 10.1007/s10067-016-3353-3 27448150

[4] Clauw DJ. Fibromyalgia: a clinical review. JAMA (2014) 311:1547–55. 10.1001/jama.2014.3266 24737367

[5] Vincent A Lahr BD Wolfe F Clauw DJ Whipple MO Oh TH. Prevalence of fibromyalgia: a population-based study in Olmsted County, Minnesota, utilizing the Rochester epidemiology project. Arthritis Care Res. (2013) 65:786–92. 10.1002/acr.21896 23203795 PMC3935235

[6] Haliloglu S Carlioglu A Akdeniz D Karaaslan Y Kosar A. Fibromyalgia in patients with other rheumatic diseases: prevalence and relationship with disease activity. Rheumatol Int. (2014) 34:1275–80. 10.1007/s00296-014-2972-8 24589726

[7] Duffield SJ Miller N Zhao S Goodson NJ. Concomitant fibromyalgia complicating chronic inflammatory arthritis: a systematic review and meta-analysis. Rheumatology (Oxford) (2018) 57:1453–60. 10.1093/rheumatology/key112.ory 29788461 PMC6055651

[8] Santiago MG Marques A Kool M Geenen R da Silva JAP. Invalidation in patients with rheumatic diseases: clinical and psychological framework. J Rheumatol. (2017) 44:512–8. 10.3899/jrheum.160559 28202742

[9] Hench PK Nonarticular rheumatism, 22nd rheumatism review: review of the American and English literature for the years 1973 and 1974. Arthritis Rheum. (1976) 19:1081–9. 793600

[10] Wolfe F Smythe HA Yunus MB Bennett RM Bombardier C Goldenberg DL. The American College of Rheumatology criteria for the classification of fibromyalgia. Arthritis Rheum. (1990) 33:160–72. 2306288 10.1002/art.1780330203

[11] MacFarlane GJ Croft PR Schollum AJ. Widespread pain: is an improved classification possible? J Rheumatol. (1996) 23:1628–32. 8877936

[12] Wolfe F. Pain extent and diagnosis: development and validation of the regional pain scale in 12,799 patients with rheumatic disease. J Rheumatol. (2003):369–78. 12563698

[13] Wolfe F Michaud K. Severe rheumatoid arthritis (RA), worse outcomes, comorbid illness, and sociodemographic disadvantage characterize ra patients with fibromyalgia. J Rheumatol. (2004) 31:695–700. 15088293

[14] Wolfe F Clauw DJ Fitzcharles MA Goldenberg DL Katz RS Mease P. The American College of Rheumatology preliminary diagnostic criteria for fibromyalgia and measurement of symptom severity. Arthritis Care Res. (2010) 62:600–10. 10.1002/acr.20140 20461783

[15] Wolfe F Clauw DJ Fitzcharles MA Goldenberg DL Häuser W Katz RS. Fibromyalgia criteria and severity scales for clinical and epidemiological studies: a modification of the ACR preliminary diagnostic criteria for fibromyalgia. J Rheumatol. (2011) 38:1113–22. 10.3899/jrheum.100594 21285161

[16] Wolfe F Clauw DJ Fitzcharles MA Goldenberg DL Häuser W Katz RL. 2016 Revisions to the 2010/2011 fibromyalgia diagnostic criteria. Semin Arthritis Rheum. (2016) 46:319–29. 10.1016/j.semarthrit.2016.08.012 27916278

[17] Perrot S Bouhassira D Fermanian J. Development and validation of the Fibromyalgia Rapid Screening Tool (FiRST). Pain (2010) 150:250–6. 10.1016/j.pain.2010.03.034 20488620

[18] White KP Speechley M Harth M Ostbye T White KP Speechley M. The London Fibromyalgia Epidemiology Study: the prevalence of fibromyalgia syndrome in London, Ontario. J Rheumatol. (1999) 26:1570–6. 10405947

[19] Akgul O Ozgocmen S. Classification criteria for spondyloarthropathies. World J Orthop. (2011) 2:107 10.5312/wjo.v2.i12.107 22474629 PMC3302034

[20] Bowness P HLA B-27. Annu Rev Immunol. (2015) 33:29–48. 10.1146/annurev-immunol-032414-112110 25861975

[21] Reveille JD Hirsch R Dillon CF Carroll MD Weisman MH. The prevalence of HLA-B27 in the US: data from the US National Health and Nutrition Examination Survey, 2009. Arthritis Rheum. (2012) 64:1407–11. 10.1002/art.33503 22139851 PMC4038331

[22] Omair MA AlDuraibi FK Bedaiwi MK Abdulaziz S Husain W El Dessougi M. Prevalence of HLA-B27 in the general population and in patients with axial spondyloarthritis in Saudi Arabia. Clin Rheumatol. (2017) 36:1537–43. 10.1007/s10067-017-3655-0 28456926

[23] Calin A Porta J Fries JF Schurman DJ. Clinical history as a screening test for ankylosing spondylitis. JAMA (1977) 237:2613–4. 140252

[24] Rudwaleit M Khan MA Sieper J. The challenge of diagnosis and classification in early ankylosing spondylitis: do we need new criteria? Arthritis Rheum. (2005) 52:1000–8. 10.1002/art.20990 15818678

[25] Rudwaleit M Metter A Listing J Sieper J Braun J. Inflammatory back pain in ankylosing spondylitis: a reassessment of the clinical history for application as classification and diagnostic criteria. Arthritis Rheum. (2006) 54:569–78. 10.1002/art.21619 16447233

[26] Rudwaleit M van der Heijde D Landewé R Listing J Akkoc N Brandt J. The development of Assessment of Spondyloarthritis International Society classification criteria for axial spondyloarthritis (part II): validation and final selection. Ann Rheum Dis. (2009) 68:777–83. 10.1136/ard.2009.108233 19297344

[27] Erlich GE Population studies of the rheumatic diseases. Arch Intern Med. (1969) 124:126–7. 10.1001/archinte.1969.00300170128048

[28] van der Linden S Valkenburg HA Cats A Evaluation of diagnostic criteria for ankylosing spondylitis. A proposal for modification of the New York criteria. Arthritis Rheum. (1984) 27:361–8. 6231933 10.1002/art.1780270401

[29] Amor B Dougados M Mijiyawa M. Criteria of the classification of spondylarthropathies. Rev Rhum Mal Osteoartic. (1990) 57:85–9. 2181618

[30] Dougados M van der Linden S Juhlin R Huitfeldt B Amor B Calin A. The European Spondylarthropathy Study Group preliminary criteria for the classification of spondylarthropathy. Arthritis Rheum. (1991) 34:1218–27. 1930310 10.1002/art.1780341003

[31] Taylor W Gladman D Helliwell P Marchesoni A Mease P Mielants H CASPAR Study Group. Classification criteria for psoriatic arthritis: development of new criteria from a large international study. Arthritis Rheum. (2006) 54:2665–73. 10.1002/art.21972 16871531

[32] Baraliakos X Braun J. Non-radiographic axial spondyloarthritis and ankylosing spondylitis: what are the similarities and differences? RMD Open (2015) 1:e000053. 10.1136/rmdopen-2015-000053 26557375 PMC4632143

[33] Lambert RG Bakker PA van der Heijde D Weber U Rudwaleit M Hermann KG. Defining active sacroiliitis on MRI for classification of axial spondyloarthritis: update by the ASAS MRI working group. Ann Rheum Dis. (2016) 75:1958–63. 10.1136/annrheumdis-2015-208642 26768408

[34] Aloush V Ablin JN Reitblat T Caspi D Elkayam O. Fibromyalgia in women with ankylosing spondylitis. Rheumatol Int. (2007) 27:865–8 10.1007/s00296-007-0344-3 17476510

[35] Almodóvar R Carmona L Zarco P Collantes E González C Mulero J. Fibromyalgia in patients with ankylosing spondylitis: prevalence and utility of the measures of activity, function and radiological damage. Clin Exp Rheumatol. (2010) 28(6 Suppl. 63):S33–9. 21176420

[36] Azevedo VF Paiva Edos S Felippe LR Moreira RA. Occurrence of fibromyalgia in patients with ankylosing spondylitis. Rev Bras Reumatol. (2010) 50:646–50. 10.1590/S0482-50042010000600005 21243305

[37] Husted JA Tom BD Farewell VT Gladman DD. Longitudinal analysis of fatigue in psoriatic arthritis. J Rheumatol. (2010) 37:1878–84. 10.3899/jrheum.100179 20595286

[38] Roussou E Ciurtin C. Clinical overlap between fibromyalgia tender points and enthesitis sites in patients with spondyloarthritis who present with inflammatory back pain. Clin Exp Rheumatol. (2012) 30 (Suppl. 74):S24–30. 22935170

[39] Husted JA Thavaneswaran A Chandran V Gladman DD. Incremental effects of comorbidity on quality of life in patients with psoriatic arthritis. J Rheumatol. (2013) 40:1349–56. 10.3899/jrheum.121500 23772076

[40] Demirdal S Çakir T Tugrul T Subaşi V Coexisting of fibromyalgia syndrome and ankylosing spondylitis. Acta Med Mediterr. (2013) 29:827.

[41] Salaffi F De Angelis R Carotti M Gutierrez M Sarzi-Puttini P Atzeni F. Fibromyalgia in patients with axial spondyloarthritis: epidemiological profile and effect on measures of disease activity. Rheumatol Int. (2014) 34:1103–10. 10.1007/s00296-014-2955-9 24509896

[42] Graceffa D Maiani E Sperduti I Ceralli F Bonifati C. Clinical remission of psoriatic arthritis in patients receiving continuous biological therapies for 1 year: the experience of an outpatient dermatological clinic for psoriasis. Clin Exp Dermatol. (2015) 40:136–41. 10.1111/ced.12504 25438647

[43] Bello N Etcheto A Béal C Dougados M Moltó A. Evaluation of the impact of fibromyalgia in disease activity and treatment effect in spondyloarthritis. Arthritis Res Ther. (2016) 18:42. 10.1186/s13075-016-0943-z 26860612 PMC4748456

[44] Wach J Letroublon MC Coury F Tebib JG. Fibromyalgia in spondyloarthritis: effect on disease activity assessment in clinical practice. J Rheumatol. (2016) 43:2056–63. 10.3899/jrheum.160104 27633820

[45] Brikman S Furer V Wollman J Borok S Matz H Polachek A. The effect of the presence of fibromyalgia on common clinical disease activity indices in patients with psoriatic arthritis: a cross-sectional study. J Rheumatol. (2016) 43:1749–54. 10.3899/jrheum.151491 27252430

[46] Fan A Tournadre A Pereira B Tatar Z Couderc M Malochet-Guinamand S. Performance of Fibromyalgia Rapid Screening Tool (FiRST) to detect fibromyalgia syndrome in rheumatic diseases. Rheumatology (Oxford) (2016) 55:1746–50. 10.1093/rheumatology/kew244 27313278

[47] Macfarlane GJ Barnish MS Pathan E Martin KR Haywood KL Siebert S. Co-occurrence and characteristics of patients with axial spondyloarthritis who meet criteria for fibromyalgia: results from a UK National Register. Arthritis Rheumatol. (2017) 69:2144–2150. 10.1002/art.40185 28622461

[48] Baraliakos X Regel A Kiltz U Menne HJ Dybowski F Igelmann M Patients with fibromyalgia rarely fulfil classification criteria for axial spondyloarthritis. Rheumatology (Oxford) (2017) 5:1541–7. 10.1093/rheumatology/kex318 28968885

[49] Di Carlo M Becciolini A Lato V Crotti C Favalli EG Salaffi F The 12-item psoriatic arthritis impact of disease questionnaire: construct validity, reliability, and interpretability in a clinical setting. J Rheumatol. (2017) 44:279–85. 10.3899/jrheum.160924 27909086

[50] Fan A Pereira B Tournadre A Tatar Z Malochet-Guinamand S Mathieu S. Frequency of concomitant fibromyalgia in rheumatic diseases: monocentric study of 691 patients. Semin Arthritis Rheum. (2017) 47:129–32. 10.1016/j.semarthrit.2017.01.005 28216193

[51] Dantu A Michaud J Prieur MG Da Silva F Pouplin S Lequerre T AB0923 meeting the fibromyalgia criteria has a negative impact on tnf inhibitors efficacy in patients with axial spondyloarthritis. Ann Rheum Dis. (2017) 76:1378 10.1136/annrheumdis-2017-eular.4310

[52] Dantu A Michaud J Bréhier Q Banse C Avenel G Lequerré T. Impact of ACR 2010 fibromyalgia criteria fulfillment on disease activity evaluation in patients with axial spondyloarthritis treated with infliximab. Joint Bone Spine (2018). [Epub ahead of print]. 10.1016/j.jbspin.2018.04.001. 29680208

[53] Moltó A Etcheto A Gossec L Boudersa N Claudepierre P Roux N. Evaluation of the impact of concomitant fibromyalgia on TNF alpha blockers' effectiveness in axial spondyloarthritis: results of a prospective, multicentre study. Ann Rheum Dis. (2018) 77:533–40 10.1136/annrheumdis-2017-212378 29183878

[54] Kaşkari D Yücel AE Agildere M. The prevalence of spondyloarthropathy in fibromyalgia patients. Mod Rheumatol. (2017) 27:875–80. 10.1080/14397595.2016.1265694 27919196

[55] Roussou E Georgiou A. Assessment of the current inflammatory back pain criteria in patients with fibromyalgia. Clin Exp Rheumatol. (2017) 35:S132–3. 28281455

[56] Ablin JN Eshed I Berman M Aloush V Wigler I Caspi D. Prevalence of axial spondyloarthritis among patients with fibromyalgia: a magnetic resonance imaging study with application of the Assessment of Spondyloarthritis International Society classification criteria. Arthritis Care Res (Hoboken). (2017) 69:724–9. 10.1002/acr.22967 27390225

[57] Günaydin R Göksel Karatepe A Ceşmeli N Kaya T. Fatigue in patients with ankylosing spondylitis: relationships with disease-specific variables, depression, and sleep disturbance. Clin Rheumatol. (2009) 28:1045–51. 10.1007/s10067-009-1204-1 19504231

[58] Ghosh N Ruderman EM. Nonradiographic axial spondyloarthritis: clinical and therapeutic relevance. Arthritis Res Ther. (2017) 19:286 10.1186/s13075-017-1493-8 29273055 PMC5741895

[59] Watad A Cuthbert RJ Amital H McGonagle D. Enthesitis: much more than focal insertion point inflammation. Curr Rheumatol Rep. (2018) 20:41 10.1007/s11926-018-0751-3 29846815 PMC5976708

[60] Schett G Lories RJ D'Agostino MA Elewaut D Kirkham B Soriano ER. Enthesitis: from pathophysiology to treatment. Nat Rev Rheumatol. (2017) 13:731–41. 10.1038/nrrheum.2017.188 29158573

[61] Marchesoni A De Marco G Merashli M McKenna F Tinazzi I Marzo-Ortega H. The problem in differentiation between psoriatic-related polyenthesitis and fibromyalgia. Rheumatology (Oxford) (2018) 57:32–40. 10.1093/rheumatology/kex079 28387854

[62] Dougados M Logeart I Szumski A Coindreau J Jones H. Evaluation of whether extremely high enthesitis or bath ankylosing spondylitis disease activity index (BASDAI) scores suggest fibromyalgia and confound the anti-TNF response in early non-radiographic axial spondyloarthritis. Clin Exp Rheumatol. (2017) 35 Suppl. 105:50–53. 28240587

[63] Ory PA Gladman DD Mease PJ Psoriatic arthritis and imaging. Ann Rheum Dis. (2005) 64(Suppl. 2):ii55–7. 10.1136/ard.2004.033928 PMC1766871 15708938

[64] D'Agostino MA Terslev L. Imaging evaluation of the entheses: ultrasonography, MRI, and scoring of evaluation. Rheum Dis Clin North Am. (2016) 42:679–93. 10.1016/j.rdc.2016.07.012 27742021

[65] Mandl P Navarro-Compán V Terslev L Aegerter P van der Heijde D D'Agostino MA, European League Against Rheumatism (EULAR). EULAR recommendations for the use of imaging in the diagnosis and management of spondyloarthritis in clinical practice. Ann Rheum Dis. (2015) 74:1327–39. 10.1136/annrheumdis-2014-206971 25837448

[66] Khmelinskii N Regel A Baraliakos X. The role of imaging in diagnosing axial spondyloarthritis. Front Med (Lausanne) (2018) 5:106. 10.3389/fmed.2018.00106 29719835 PMC5913283

[67] Taniguchi Y Arii K Kumon Y Fukumoto M Ohnishi T Horino T. Positron emission tomography/computed tomography: a clinical tool for evaluation of enthesitis in patients with spondyloarthritides. Rheumatology (Oxford) (2010) 49:348–54. 10.1093/rheumatology/kep379 20007287

[68] Buchbender C Ostendorf B Ruhlmann V Heusch P Miese F Beiderwellen K. Hybrid 18F-labeled fluoride positron emission tomography/magnetic resonance (MR) imaging of the sacroiliac joints and the spine in patients with axial spondyloarthritis: a pilot study exploring the link of MR bone pathologies and increased osteoblastic activity. J Rheumatol. (2015) 42:1631–7. 10.3899/jrheum.150250 26136491

[69] D'Agostino MA Aegerter P Bechara K Salliot C Judet O Chimenti MS. How to diagnose spondyloarthritis early. Accuracy of peripheral enthesitis detection by power Doppler ultrasonography. Ann Rheum Dis. (2011) 70:1433–40. 10.1136/ard.2010.138701 21586438

[70] de Miguel E Munoz-Fernandez S Castillo C Cobo-Ibáñez T Martín-Mola E Diagnostic accuracy of enthesis ultrasound in the diagnosis of early spondyloarthritis. Ann Rheum Dis. (2011) 70:434–9. 10.1136/ard.2010.134965 21131646

[71] Ash ZR Tinazzi I Gallego CC Kwok C Wilson C Goodfield M. Psoriasis patients with nail disease have a greater magnitude of underlying systemic subclinical enthesopathy than those with normal nails. Ann Rheum Dis. (2012) 71:553–6. 10.1136/annrheumdis-2011-200478 22156725

[72] Aydin SZ Ash ZR Tinazzi I Castillo-Gallego C Kwok C Wilson C. The link between enthesitis and arthritis in psoriatic arthritis: a switch to a vascular phenotype at insertions may play a role in arthritis development. Ann Rheum Dis. (2013) 72:992–5. 10.1136/annrheumdis-2012-201617 22863575

[73] Marchesoni A De Lucia O Rotunno L De Marco G Manara M. Entheseal power Doppler ultrasonography: a comparison of psoriatic arthritis and fibromyalgia. J Rheumatol Suppl. (2012) 89:29–31. 10.3899/jrheum.120238 22751587

[74] Godfrin B Zabraniecki L Lamboley V Bertrand-Latour F Sans N Fournié B. Spondyloarthropathy with entheseal pain. A prospective study in 33 patients. Joint Bone Spine (2004) 71:557–62. 10.1016/j.jbspin.2003.10.010 15589439

[75] Østergaard M Eshed I Althoff CE Poggenborg RP Diekhoff T Krabbe S. Whole-body magnetic resonance imaging in inflammatory arthritis: systematic literature review and first steps toward standardization and an OMERACT scoring system. J Rheumatol. (2017) 44:1699–705. 10.3899/jrheum.161114 28620061

[76] Weckbach S Schewe S Michaely HJ Steffinger D Reiser MF Glaser C. Whole-body MR imaging in psoriatic arthritis: additional value for therapeutic decision making. Eur J Radiol. (2011) 77:149–55. 10.1016/j.ejrad.2009.06.020 19632076

[77] Poggenborg RP Eshed I Østergaard M Sørensen IJ Møller JM Madsen OR. Enthesitis in patients with psoriatic arthritis, axial spondyloarthritis and healthy subjects assessed by ‘head-to-toe’ whole-body MRI and clinical examination. Ann Rheum Dis. (2015) 74:823–9. 10.1136/annrheumdis-2013-204239 24389294

[78] Rudwaleit M. Fibromyalgia is not axial spondyloarthritis: towards an appropriate use of the ASAS classification criteria for axial SpA. Rheumatology (Oxford) (2017) 57:1510–2. 10.1093/rheumatology/kex372 29045716

